# Dissecting the immune discrepancies in mouse liver allograft tolerance and heart/kidney allograft rejection

**DOI:** 10.1111/cpr.13555

**Published:** 2023-09-25

**Authors:** Jun Pan, Fang Ye, Hui Li, Chengxuan Yu, Jiajia Mao, Yanyu Xiao, Haide Chen, Junqing Wu, Jiaqi Li, Lijiang Fei, Yijun Wu, Xiaoming Meng, Guoji Guo, Yingying Wang

**Affiliations:** ^1^ Department of Thyroid Surgery, the First Affiliated Hospital, School of Medicine Zhejiang University Hangzhou China; ^2^ Liangzhu Laboratory Zhejiang University Hangzhou China; ^3^ Center for Stem Cell and Regenerative Medicine and Bone Marrow Transplantation Center of the First Affiliated Hospital Zhejiang University School of Medicine Hangzhou China; ^4^ Key Laboratory of Combined Multiorgan Transplantation, Ministry of Public Health, State Key Laboratory for Diagnosis and Treatment of Infectious Diseases, Division of Hepatobiliary and Pancreatic Surgery, The First Affiliated Hospital, School of Medicine Zhejiang University Hangzhou China; ^5^ Kidney Disease Center, The First Affiliated Hospital, School of Medicine Zhejiang University Hangzhou China; ^6^ Inflammation and Immune Mediated Diseases Laboratory of Anhui Province, Anhui Institute of Innovative Drugs, School of Pharmacy Anhui Medical University, The Key Laboratory of Anti‐inflammatory of Immune Medicines, Ministry of Education Hefei China; ^7^ Zhejiang Provincial Key Lab for Tissue Engineering and Regenerative Medicine Dr. Li Dak Sum & Yip Yio Chin Center for Stem Cell and Regenerative Medicine Hangzhou Zhejiang China

## Abstract

The liver is the most tolerogenic of transplanted organs. However, the mechanisms underlying liver transplant tolerance are not well understood. The comparison between liver transplantation tolerance and heart/kidney transplantation rejection will deepen our understanding of tolerance and rejection in solid organs. Here, we built a mouse model of liver, heart and kidney allograft and performed single‐cell RNA sequencing of 66,393 cells to describe the cell composition and immune cell interactions at the early stage of tolerance or rejection. We also performed bulk RNA‐seq of mouse liver allografts from Day 7 to Day 60 post‐transplantation to map the dynamic transcriptional variation in spontaneous tolerance. The transcriptome of lymphocytes and myeloid cells were characterized and compared in three types of organ allografts. Cell–cell interaction networks reveal the coordinated function of Kupffer cells, macrophages and their associated metabolic processes, including insulin receptor signalling and oxidative phosphorylation in tolerance induction. *Cd11b*+ dendritic cells (DCs) in liver allografts were found to inhibit cytotoxic T cells by secreting anti‐inflammatory cytokines such as *Il10*. In summary, we profiled single‐cell transcriptome analysis of mouse solid organ allografts. We characterized the immune microenvironment of mouse organ allografts in the acute rejection state (heart, kidney) and tolerance state (liver).

## Graphical abstract

Here, we established mouse models of liver, heart and kidney transplantation. We performed single‐cell analysis of the allografts at the early phase of rejection/tolerance as well as bulk RNA analysis of long‐term tolerance in liver allograft at different time point. We compared gene expression patterns of the immune microenvironment in organ allografts. We identified coordinated function of macrophages and associated metabolic process including insulin receptor signaling and oxidative phosphorylation process in liver transplantation tolerance induction.

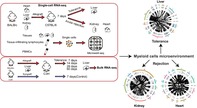

## 1 INTRODUCTION

Around 120,000 new organ transplantations were performed each year. But limited organ supply and the lack of improvement in long‐term allograft survival during the past few decades resulted in suboptimal outcomes, with only 1 million persons worldwide obtaining functioning solid‐organ transplants.^1^ The liver is considered the most tolerogenic of transplanted organs. Approximately 20% of the stable liver transplant recipients can be weaned safely off all immunosuppression.^2^ Although some insights have been gained into how the local microenvironment and specific molecular pathways promote donor‐specific tolerance, the mechanisms underlying liver transplant tolerance are not well studied.^3^

Rejection and tolerance involve the complex interaction of different immune and parenchymal cells. Orthotopic liver transplantation in mice is considered a spontaneous immunogenic tolerance transplantation model, while heart and kidney transplantation model need immunosuppressive drug administration for longer allograft survival.^4^ The setup of mouse liver, heart and kidney transplantation is a technically demanding surgical procedure. Our group has successfully established liver, heart and kidney transplantation models in mice to explore the molecular mechanisms of rejection or tolerance in organ transplantation.^5^, ^6^

Single cell RNA sequencing (scRNA‐seq) is able to dissect the immune microenvironment in organ transplantation.^7^ High‐throughput scRNA‐seq offer an unprecedented opportunity to comprehensively decipher the cellular heterogeneity in health and disease.^8^ Recently, scRNA‐seq has been applied to study the renal allograft rejection and transplanted liver.^9^, ^10^ In this work, we further mapped the single cell transcriptome landscape of mouse liver, heart and kidney allografts at day 7 post‐transplantation. We showed intercellular communication between myeloid cells, lymphocytes and their regulatory networks in acute rejection allografts (heart, kidney) and tolerance allografts (liver). The transcriptome of peripheral blood mononuclear cells (PBMCs) showed high similarity with liver‐infiltrating immune cells in recipient mice. To further explore the dynamic transcriptional changes in long‐term liver transplantation, we performed bulk RNA‐seq of mouse liver allografts from day 7 to day 60 post‐transplantation. Differentially expressed genes (DEGs) in bulk RNA‐seq datasets of allografted liver at day 7 post‐transplantation demonstrated high correlation with single cell datasets. This study provided molecular insights into the acute rejections that are specific to heart and kidney allografts and tolerance induction in liver allografts.

## 2 MATERIALS AND METHODS

### 2.1 The construction of mouse liver, heart and kidney transplant models

For scRNA‐seq, livers, hearts and kidneys from BALB/c mice (H‐2 d; 8 weeks old) were transplanted into C57Bl6/J recipients (8 weeks old). Mouse models of orthotopic liver transplantation, heterotopic heart transplantation and heterotopic kidney transplantation were generated as previously described.^11^, ^12^, ^13^ In brief, setup of the orthotopic liver transplantation requires efficient removal of the recipient liver and implantation of the donor liver within 20–30 min, which includes the portal vein (PV), the suprahepatic inferior vena cava (SHIVC), the infrahepatic inferior vena cava (IHIVC), and the bile duct anastomoses. In heart transplantation, the donated cardiac artery and vein were anastomosed to the recipient's abdominal aorta and vena cava, respectively. In kidney transplantation, the donated renal artery and vein were anastomosed to the recipient's abdominal aorta and vena cava, with the donated ureter attached to the recipient's bladder. The liver allograft was placed ‘orthotopically’, while the native heart or kidneys of the recipient mouse were left in place. For observation of the native and spontaneous immune response in allograft rejection or tolerance, mice were not administered immunosuppressive drugs, and euthanized on day 7 post‐transplantation. A chronic tolerance model of mouse liver transplantation was created by transplanting livers from BALB/c mice (8 weeks old) into C3H (8 weeks old) recipients, which were kept for 7 days, 15 days, 30 days and 60 days without immunosuppressive drugs until being euthanized. The experimental rats were individually caged at 21°C, exposed to a 12 h/12 h light/dark cycle, and fed sterilized water and standard rat chow. All animals received humane care, and the study was conducted in accordance with the Guide for the Care and Use of Laboratory Animals. The study was also approved by the Institutional Review Board, consistent with the Animal Protection Act of China.

### 2.2 Histology and immunostaining

The mouse liver, heart and kidney allografts were fixed overnight in 10% neutral‐buffered formalin. They were then transferred to 60% ethanol followed by dehydration in alcohol, immersion in chloroform and embedment in paraffin wax. Five‐micron sections were made and stained with histochemical dyes for haematoxylin and eosin staining (black/pink, nuclei/tissue respectively) and Masson's trichrome staining (blue/black/red, collagen/nuclei/cytoplasm respectively). For immunostaining, tissue samples were fixed with 4% formaldehyde (Thermo Fisher), cryoprotected with 30% sucrose and cut into 7 mm thick sections. For immunostaining, the following primary antibodies were used: rabbit anti‐mouse CD8 (Cell Signaling Technology, 98941,1:200) and Alexa Fluor 488 rat anti‐mouse CD11b (Abcam, ab197701, 1:100). The secondary antibody used was anti‐rabbit Alexa Fluor 594 (Cell Signaling Technology, 8889, 1:200). After the first primary antibody staining, an additional blocking step was included prior to the addition of a second primary antibody. The slides were washed again three times with 1% PBS (5–10 min) each before being mounted with ProLong® Gold Anti‐fade Reagent with DAPI (Invitrogen). The antigenic binding sites were visualized using a digital slide scanner and slide viewer software (Pannoramic 250 and Case Viewer 2.3, 3D Histech, Hungary).

### 2.3 Single‐cell suspension preparation

A total of nine fresh allograft tissue samples and nine blood samples for single cell sequencing were collected from two liver allograft recipients, two kidney allograft recipients and two heart allograft recipients sacrificed on day 7 after transplantation. Fresh allograft tissues were then digested using type II collagenase in PBS. The dissociated single cells were centrifuged, and cell pellets were resuspended in RPMI 1640 (Thermo Fisher) plus 0.04% bovine serum albumin (Sigma–Aldrich). Tissue‐infiltrating lymphocytes of the nine fresh allograft tissue samples were isolated by Ficoll‐Paque density gradient centrifugation. Viability was confirmed to be >90% in all samples via trypan blue (Thermo Fisher) staining, and the cell suspensions were kept on ice for single cell RNA‐seq.

### 2.4 Single‐cell RNA‐seq library preparation and sequencing

We performed Microwell‐seq of single cell suspensions from different samples as previously described.^14^ In brief, single cell suspensions and barcode beads were loaded into agarose microwell arrays. Beads and cells were trapped in separated microwells. Transcripts from lysed cells were captured by barcode oligodT beads. Beads were collected in a 1.5 mL tube for template switching, reverse transcription, exonuclease I treatment and cDNA amplification. Purified cDNA libraries were fragmented using a customized transposase to enrich the 3′ ends of transcripts (TruePrep DNA Library Prep Kit V2 for Illumina, Vazyme,cat #TD512). The concentrations of the DNA libraries were measured using Qubit3.0, and the fragment sizes of the libraries were analysed using an Agilent 2100 bioanalyzer. All the DNA libraries were sequenced on an Illumina HiSeq Xten in Pair‐end 150 bp mode.

### 2.5 Bulk RNA‐seq and microarray analysis

Samples of 12 liver allografts (three replicates on day 7, day 15, day 28 and day 60 post‐operation, respectively) were collected and stored at −80°C for bulk RNA sequencing. Total RNA, including miRNA from RNAlater (Ambion)‐immersed liver grafts, was isolated using the RNeasy Mini Kit and miRNeasy kit (Qiagen), according to the manufacturer's protocol. The quality and quantity of the RNA were evaluated using a NanoDrop 1000 (Thermo Fisher) and Agilent 2100 Bioanalyzer (Agilent Technologies).

Total RNA was amplified and transcribed into fluorescent cRNA using a Low Input Quick Amp Labeling Kit, One‐Color (Agilent Technologies). The labelled cRNA was purified using a RNeasy Mini Kit (Qiagen). Probe synthesis and hybridization to mRNA Microarray v4.0 (Agilent Technologies) were performed by a Gene Expression Hybridization Kit (Agilent Technologies) in a hybridization oven (Agilent Technologies), according to the manufacturer's instructions. Images of hybridized microarrays were scanned using an Agilent Scanner (Agilent Technologies), and the raw data were normalized by quantile algorithm, Gene Spring Software 11.0 (Agilent Technologies).

### 2.6 RNA‐seq data processing

We used the Drop‐seq core computational tool to preprocess the Microwell‐seq raw data, as described in the Drop‐seq computational cookbook.^15^ Filtered reads were used to identify cellular barcodes and unique molecular identifiers (UMIs). We discarded the paired reads if the quality of any base in the barcode was below 10. We used STAR (version 2.5.2a) with default parameters for mapping.^16^ Reads from mouse organ allograft data were aligned to the mouse GRCm38.88 genome. All multiple‐aligned reads were removed and GTF annotation files from GENCODE were used to tag aligned reads. For UMI count, molecular barcodes with one edit distance were merged to one within a gene. We excluded cells with fewer than 500 UMIs. A high proportion (>10%) of transcript counts derived from mitochondria‐encoded genes may indicate low cell quality. We removed these unqualified cells before downstream analysis. After obtaining the digital gene expression (DGE) data matrix, we used Seurat for downstream analysis.^17^

### 2.7 Cross‐tissue regulatory network comparison of grafted liver and normal liver

We used SCENIC^18^ to compare the gene regulatory networks between allografted and normal mouse liver. We combined the AUCell score matrices with the orthologous TFs. The TF modules were identified based on the connection specificity index (CSI).^19^ The regulatory networks were constructed based on the TF modules and visualized with a heatmap. To demonstrate the functions enriched in each module, we performed gene ontology enrichment analysis for these modules.

### 2.8 Weighted correlation network (WGCNA) analysis

Correlation network analysis of liver allografts was performed using WGCNA with default parameters, and external biological traits were assigned to related modules.^20^ To reduce the impact of data sparsity in low coverage sequencing datasets, we used pseudocells to aggregate data in the same cell type.

### 2.9 RNA velocity analysis

We used Velocity to calculate RNA velocity on the grafted T‐cell and macrophage dataset in liver allografts.^21^ This method estimates the rate of transcriptional changes of each cell based on the ratio of spliced and unspliced reads with default parameters. The loom file from the last step was treated as input. Cells that either did not have enough UMIs after the new mapping, or they did not have unspliced reads were discarded. The plot was visualized with t‐SNE embedding and the differentiation start and end points were estimated using a Markov process. We mainly followed the steps from the opened repository (https://github.com/rajewsky-lab/planarian_lineage).

### 2.10 Ligand–receptor analysis

Analysis of potential receptor–ligand pairs was performed by applying iTALK.^22^ We aggregated the gene expression levels from immune cell clusters in the allografted liver, kidney and heart. To remove the effect of cell numbers in each cluster, we selected randomly sampled pseudocells for further analysis. Receptors and ligands expressed in more than 10% of the cells in each cluster were remained. The ligand–receptor interactions were constructed as a matrix after randomly permuting 1000 cluster labels to calculate the mean expression values of the ligands and receptors. We used pairwise comparisons between all cell types to obtain a likelihood of *p* value for the filtering of false‐positive interactions. The cutoff was set with a mean expression greater than 0.1 and *p* values smaller than 0.1. We used the sum of the number of ligand–receptor pairs in each cell–cell pairs to indicate the strength of the cell–cell interactions. Finally, the interaction network was set to a degree‐sorted circle layout for visualization in Cytoscape.^23^

## 3 RESULTS

### 3.1 Single cell transcriptome landscape of mouse liver, kidney and heart allografts

**FIGURE 1:**
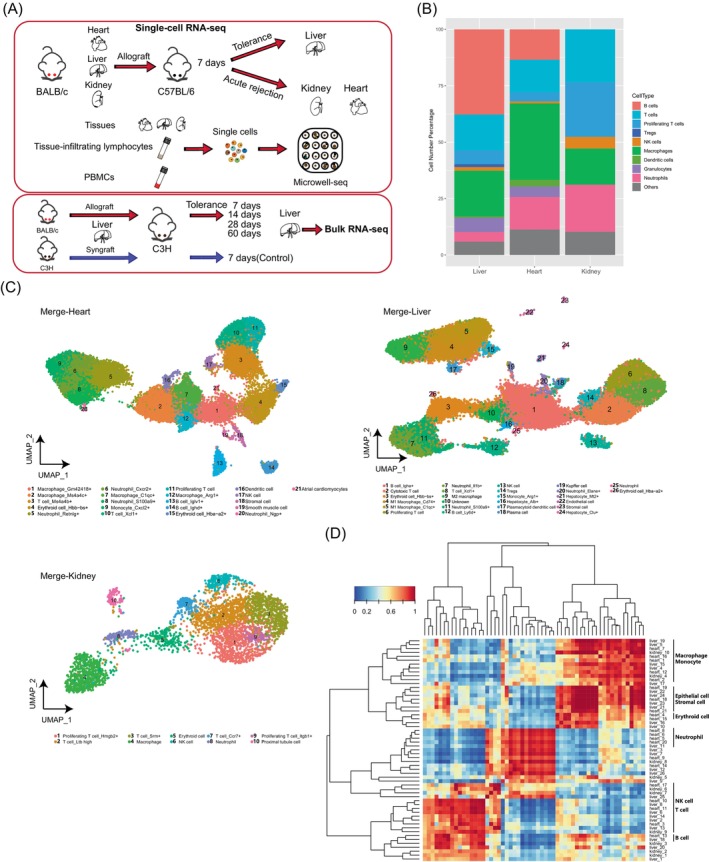
Single cell transcriptome profiling of mouse liver, heart and kidney allografts. (A) Schematic view of the workflow. (B) The fractions of different immune cells in three allografted organs. ‘Other’ represents the parenchymal cells. (C) UMAP plot showing the clusters of merged single‐cell datasets. (D) Heatmap showing the gene expression correlations of cell types in (C).

We first established mouse liver, kidney and heart allograft models. Organs from BALB/c mice were implanted into C57BL/6 recipients without any immunosuppressive treatment. On day 7 post‐transplantation, organ allografts and PBMCs in recipients were harvested (Figure 1A and Figure S1A). Haematoxylin and eosin (HE) staining indicated a certain degree of inflammatory infiltration and destruction of tissue architecture in kidney and heart (Figure S1A). Tissue‐infiltrating lymphocytes (TILs) were further harvested from three types of organs by density gradient centrifugation. We previously reported Microwell‐seq, a flexible and low‐cost high‐throughput scRNA‐seq method.^14^ Here, we adopted Microwell‐seq to characterize the single‐cell transcriptome of immune cells and nonimmune cells in three types of mouse organ allografts. PBMCs, parenchymal cells (PCs) and TILs were sequenced separately (Figure 1A). Moreover, we performed bulk RNA‐seq of liver allografts at different time points post‐transplantation (day 7, day 14, day 28 and day 60) to explore the dynamic transcriptome changes of long‐term tolerance formation (Figure 1A).

scRNA‐seq profiles generated a total of 66,393 cells that passed quality control (Figure S1B). After unsupervised clustering, single cells in PBMCs, PCs and TILs were merged and visualized using Uniform Manifold Approximation and Projection (UMAP) (Figure 1C and Figure S1C). We identified common lymphocytes and major myeloid cells based on canonical signature genes (Figure S1D). Over 80% of the single cells in the datasets were immune cells (Figure 1B).

In liver allografts, we identified 21 immune cell clusters, 3 hepatocyte clusters (Clusters 16, 21, and 24, marked by high expression of *Alb*), a stromal cell cluster (Cluster 23, marked by high expression of *Dcn* and *Mgp*) and an endothelial cell cluster (Cluster 22, marked by high expression of *Eng* and *Egfl7*) (Figure 1C and Figure S1C). In heart allografts, cells from Cluster 21 were identified as atrial cardiomyocytes based on the enrichment of *Myl7*, *Myl4* and *Acta1*. Cluster 19 was identified as smooth muscle cells with specific expression of *Acta2* and *Myl9*. Cluster 18 highly expressed stromal cell markers such as *Col3a1*, *Col1a2* and *Mgp*. In kidney allografts, we defined 9 immune cell clusters and proximal tubule cells with high expression of *Kap*.^24^

In order to compare the gene expression correlations of a cross‐tissue single‐cell transcriptome dataset, we performed MetaNeighbor analysis of cell types in different organ allografts^25^ (Figure 1D). Gene expression modules showed high similarity in parenchymal cells and immune cells between the tolerance state (liver) and acute rejection state (heart and kidney) at the early stage after allografts. Heterogeneity of myeloid cells was also observed between liver and other organs, indicating the potential perturbation of a different immune environment in liver.

### 3.2 Transcriptome profiling of lymphocytes reveals the spontaneous tolerance state in mouse liver allografts

**FIGURE 2:**
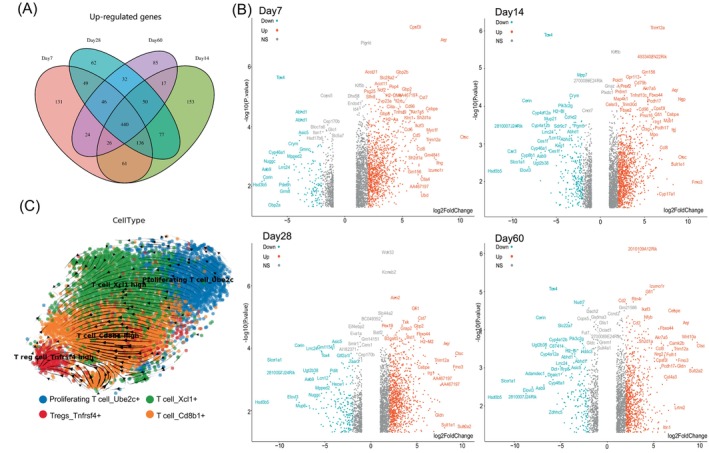
Bulk RNA‐seq analysis and T cell trajectory in liver allografts. (A) Venn plot showing the overlapped differentially expressed genes between different time points in liver allografts. (B) Volcano plot showing the differentially expressed genes in liver allografts at different stages (bulk RNA‐seq). Significantly up‐regulated and down‐regulated genes (log2FoldChange > 1.5) are labelled (NS: not significant). (C) RNA velocities of major T cell subsets in allografted liver (single‐cell RNA‐seq data).

Next, we performed differentially expressed genes (DEGs) analysis of bulk RNA‐seq datasets at different time points in liver allograft. A higher number of up‐regulated genes was observed at the early stage (day 7, day 14) (Figure 2A). We observed up‐regulation of *Ctsc*, *Cebpe*, *Ifng*, and *Ctla4* (associated with regulatory T‐cell function and activation) at day 7 post‐transplantation (Figure 2B). Gene function and KEGG pathway enrichment of up‐regulated genes showed antigen processing and presentation program, cytokine‐receptor interaction between myeloid cells and T cells (Figure S2A,B).^26^, ^27^ The function of DEGs at the late stage (day 14–day 60) revealed a growing tolerance induction process. Regulation of interferon‐gamma production and response to interferon‐gamma (interferon‐gamma‐mediated signalling pathway) were enriched at day 14 and day 28, respectively. At day 60, lymphoid and nonlymphoid cell immunoregulatory interactions, metabolic processes associated with insulin secretion and fatty acid and fat‐soluble vitamin processes were involved in long‐term tolerance induction. KEGG pathway analysis suggested that the dynamic state of Th1, Th2 and Th17 cells and myeloid cells mediated long‐term tolerance in liver allografts.^28^, ^29^

We further investigated the heterogeneity of immune cells in liver allografts. Re‐clustering of immune cell clusters identified specific marker genes of major lymphocytes and myeloid cells (Figure S3A). We identified four T‐cell subsets with different gene expression patterns (Figure S3B,C). Notably, exhausted T cells (*Pdcd1+* and *Cd160+*) suggested an unique immunosuppressive state in liver allografts.^30^ We also identified regulatory T cells (Tregs) that differentially expressing *Cd4* and *Ctla4* (Figure S3D). To infer cell dynamics of T cells in liver allograft, we used RNA velocity to evaluate the differentiation of T cell subsets.^21^ The directional flow towards Tregs and extensive expression of *Pdcd1* in all the subsets suggested the initiation of T cell dysfunction at day 7 post‐transplantation^31^ (Figure 2C). The cell‐cycle heatmap indicated that the majority of proliferating T cells expressed cell cycle genes in G2/M phase (Figure S3E).

Cross‐tissue clustering of T cells also revealed similar functional T cell subsets among liver, heart and kidney allografts (Figure S3F). Higher proportion of Tregs and proliferating T cells were observed in liver allografts (Figure S3G,H). DEGs analysis of T cells between liver allografts and the other two tissues implied specific expression of *Saa3* in liver allografts, which was associated with Th17 cell differentiation^32^ (Figure S3I).

Other lymphocytes were also defined in the allografted liver immune microenvironment. B cells with high expression of *Cd79a* and *Cd79b* were distinguished from plasma cells with unique expression of transcription factor *Xbp1*. The plasma cells were enriched in liver allografts than PBMCs (Figure S1C). Recent studies have indicated the role of plasma cells in the regulation of regulatory B cell (Breg) activity in engraftment.^33^, ^34^ Natural killer (NK) cells were defined using marker genes such as *Nkg7* and *Ly49* family receptors. NK cells were partly derived from PBMCs and most of them were conventional NK (cNK) cells.^35^

### 3.3 Analysis of myeloid cells in liver allografts revealed coordinated communication between macrophages and T cells

Analysis of myeloid cells in liver allografts provides insights into the heterogeneity of Kupffer cells and macrophages. Kupffer cells (enriched expression of *Clec4f*, *Vsig4* and *Cd68*) were liver‐resident macrophages which were distributed in the hepatic sinusoid. It has been reported that *IL‐10* and *TGF‐β* secreted by Kupffer cells could induce T cell suppression.^36^ We examined the expression of cytokine‐related ligand–receptor pairs between Kupffer cells and other immune cells in liver allografts (Figure S4A). Cytokine regulatory networks highlighted the enrichment of inflammatory associated *CXCL13‐CXCR3*, *CCL24‐CCR3*, *IL1A‐IL1R2* and *IL18‐IL18R1* axes between Kupffer cells, macrophages and lymphocytes.^37^, ^38^ The focused analysis of *IL‐10* demonstrated a ubiquitous interaction between Kupffer cells and other immune cells in maintaining immune tolerance in liver allografts^39^ (Figure S4B). The ligand–receptor pairs between Kupffer cells and Tregs showed enrichment via the *CXCL3‐CXCR3* and *CCR (CCL‐CCR)* families. These interactions suggested that Kupffer cells could trigger the recruitment of Tregs in the postischemic liver after transplantation.^40^

We further performed re‐clustering of macrophages and monocytes in liver allografts based on their original marker genes (Figure S4C). *Chil3*+ macrophages demonstrated distinct gene expression patterns compared with classically activated M1 macrophages which highly expressed MHC‐II molecules (Figure S4D). DEGs between *Chil3*+ macrophages and M1 macrophages revealed unique expression of M2a macrophages markers such as *Fn1* and *Chil3* (Figure S4E).^41^, ^42^ *Chil3* ^+^ M2a macrophages selectively express haptoglobin (*Hp*), which forms haemoglobin: haptoglobin complexes (*Hp:Hb*) that further promote haemoglobin scavenger receptor expression in M2 macrophages.^43^ In mouse heart allograft model, targeted treatment of mTOR‐dependent M2 macrophages was confirmed to prevent chronic allograft rejection via the *PD‐1/PD‐L1* coinhibitory pathway.^44^ Monocytes and the polarization of *Ly6c* ^*−*^ */Chil3* ^+^ M2a macrophages play critical roles in anti‐inflammation and tissue remodelling after organ transplantation.^45^, ^46^

Myeloid‐derived suppressor cells (MDSCs) are able to inhibit the function of infiltrated lymphocytes in liver allografts. Neutrophils play a key role in the activation of lymphocytes and antigen‐presenting cells in the adaptive immune response.^47^, ^48^ A recent study reported that neutrophil‐derived colony‐stimulating factor 1 (*Csf1*) could promote allograft tolerance by regulating the polarization and proliferation of tissue‐restricted macrophages.^49^ The number of suppressive *Ly6c* ^−^ macrophages increased under the regulation of *Csf1* after transplantation.^50^ We analysed the ligand–receptor pairs of *Csf1* in liver allografts (Figure S4F). Neutrophils in Clusters C7, C11 and C25 showed enrichment of *Csf1‐Csf1r* interactions with macrophages (Clusters C4, C5 and C9), suggesting that infiltrating neutrophils may mediate the polarization of multiple types of *Ly6c*‐ macrophages through the *Csf1‐Csf1r* axis and participate in the regulation of allograft tolerance.

### 3.4 Comparison of allografted and normal mouse liver

**FIGURE 3:**
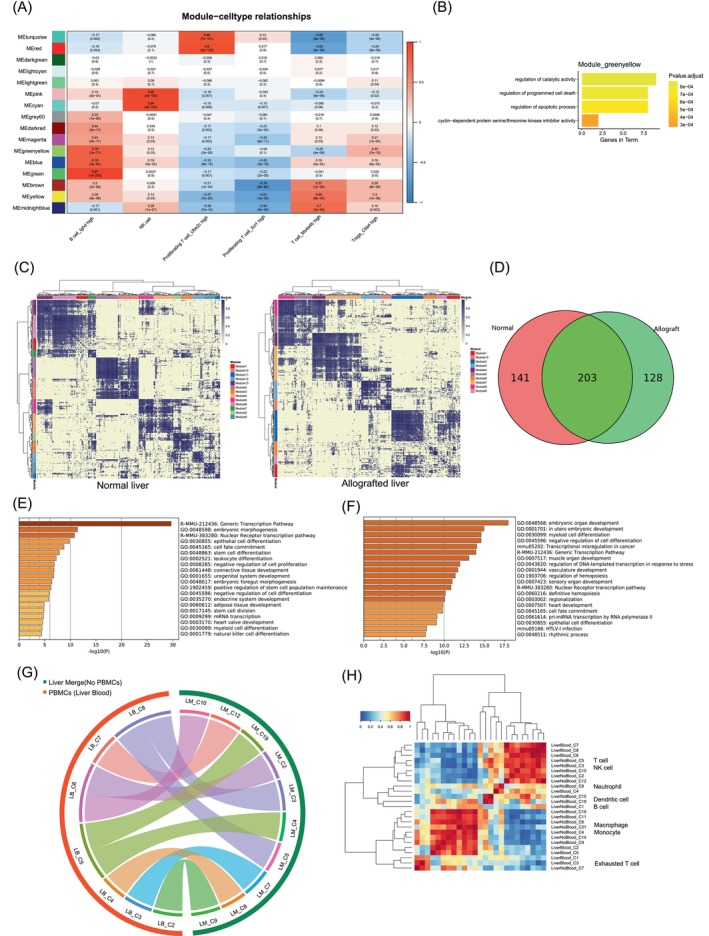
Transcription factor enrichment and correlation analysis of immune cells in normal and allografted mouse liver. (A) Gene module correlations of immune cells in allografted liver. (B) Gene Ontology enrichment of the green yellow gene module (including *Gzma*, *Iglc3*, *Foxp3* and *Tnfrsf4*). (C) Correlation of transcription factor modules in normal (left) and allografted mouse liver (right). (D) Venn plot showing the overlapping of transcription factors between normal and allografted mouse liver. (E, F) Gene Ontology enrichment of transcription factor modules in normal (E) and allografted mouse liver (F). (G) Correlation of gene expressions between PBMCs and tissue‐infiltrating immune cells (LM: clusters in tissue and tissue‐infiltrating lymphocytes, LB: clusters in PBMCs). (H) Heatmap showing the Pearson correlation values between PBMCs and tissue‐infiltrating immune cells in allografted liver.

We integrated the datasets of allografted and normal mouse livers to construct the regulatory network of liver allografts.^14^ We used coexpression network analysis (weighted gene coexpression network, WGCNA) to decipher the common gene modules^20^ (Table S1). We identified 60 gene modules (ME) with different colours (Figure S5A). T cell related gene modules containing *Klra4*, *Cd3* (module 8, pink) and *Cd4*, *Snn*, *Gngt2*, *Gpcd3* (module 35, sienna) were grouped in neighbouring clusters (Figure S5B). We calculated the correlation between gene modules and lymphocytes (Figure 3A). Modules 11 (green yellow) exhibited a high correlation with B cells and Tregs. Significant genes in this module included *Gzma*, *Iglc3*, *Foxp3* and *Tnfrsf4*. Gene ontology enrichment analysis of this module showed cyclin‐dependent protein serine/threonine kinase inhibitor activity, apoptotic process and programmed cell death (Figure 3B). Gene functions in B cells and Tregs showed a positive effect on the regulation of *CD8*+ T‐cell exhaustion.

Then, we integrated common cell types of allografted and normal mouse liver. Enrichment of transcription factors (TFs) was visualized in clustered modules (Figure 3C). The overlap of TFs suggested unique regulatory events in normal and allografted livers (Figure 3D). Gene ontology enrichment of specific TFs in normal liver indicated epithelial cell differentiation and negative regulation of cell proliferation (Figure 3E). Functional enrichment terms of TFs in liver allografts showed myeloid cell differentiation (Figure 3F). Common and specific TFs were clustered into 13 and 10 modules in allografted and normal mouse liver, respectively. Module 9 in normal liver contained *Bach2* and *Ebf1* which could promote B cell development (Figure S5C, Table S2). Of note, module 9 included Irf4, which was specifically expressed in normal liver. The deletion of *Irf4* in mice resulted in enhanced *Pd‐1* expression and *Cd4* ^+^ T‐cell dysfunction.^51^ Module 5 contained normal liver specific TFs *Batf3* and *Id2* which could cooperate with *Irf4* in dendritic cell differentiation^52^ (Figure S5D). In mouse liver allografts, module 7 contained specific TFs including *Cebpa*, *Irf8*, *Tal1*, *c‐Rel* and *Nr1h3* (Table S3). *Irf8* and *Tal1* have been shown to be involved in the maturation of monocytes and macrophages^53^, ^54^ (Figure S5E). Gene function terms of specific TFs in allografted liver T cells were involved in the mitotic cell cycle and mitochondrial function (Figure S5F). We identified potential TF correlation between *Bhlhe40* and *Cebpb* in liver allografts. The *Nr1h3‐Cebpb‐Bhlhe40* complex is required for insulin‐mediated stimulation through the dynamics of SREBP‐1c.^55^ Insulin secretion regulates insulin receptor signalling which controls T cell proliferation as well as IFNγ production.^56^ The specific TF networks suggested the dynamic metabolism regulation of T cell subsets in liver allografts.

We further questioned whether the gene expression patterns of the immune microenvironment in allografted liver could be evaluated using PBMCs at day 7 post‐transplantation. We compared the single‐cell transcriptome of PBMCs with tissue‐resident immune cells. The gene expression patterns of PBMCs and most liver‐resident immune cells exhibited a high correlation (Figure 3G). T cells and macrophages from PBMCs and liver‐resident immune cells were clustered together (Figure 3H). These results suggested that the gene expression of T cells or macrophages in PBMCs could be further explored as a novel indicator to evaluate the tolerance or rejection state in organ allografts.

### 3.5 Cross‐tissue comparison of liver, kidney and heart allografts

**FIGURE 4:**
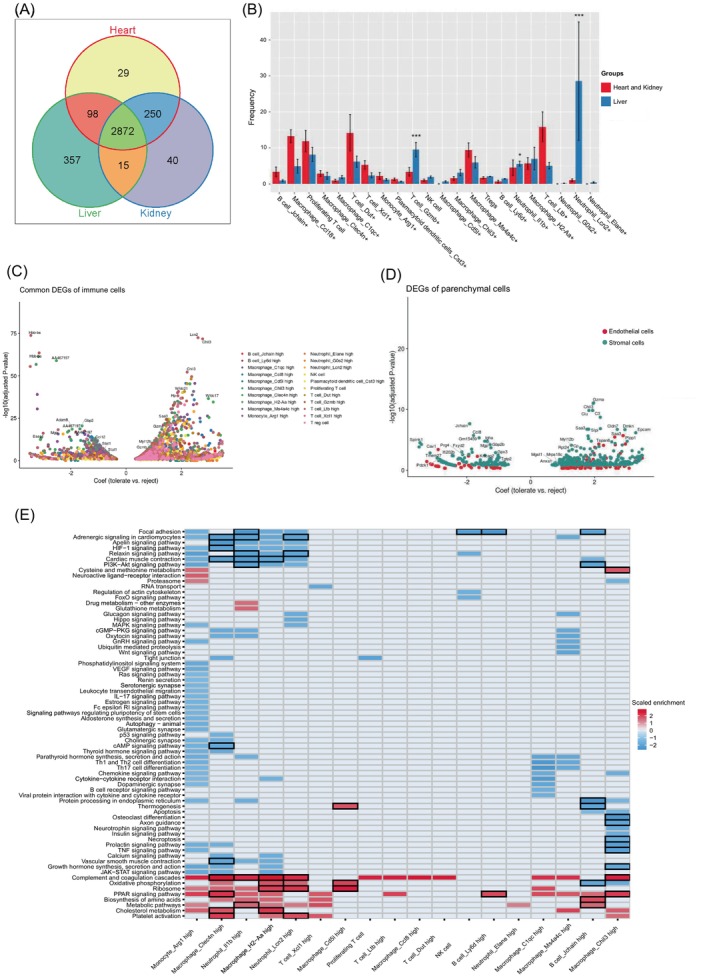
Cross‐tissue comparison between liver, heart and kidney allografts. (A) Venn plot showing the overlapped variable genes allografted liver, heart, and kidney. (B) Histogram showing the frequency comparison of immune cell between allografted liver and the other two organs. (‘***’*p* value ≤ 0.001, ‘*’*p* value ≤ 0.05). (C) Volcano plot showing the common differentially expressed genes in major immune cells between the allografted liver and the other two organs. Cell types are colour‐coded. (D) Volcano plot showing the common differentially expressed genes in endothelial cells (red dots) and stromal cells (green dots) between the allografted liver and the other two organs. (E) Heatmap showing the KEGG pathway enrichment of differentially expressed genes in major immune cells between the allografted liver and the other two organs.

The liver has the most tolerogenic properties among the three types of organ allografts. Permanent and spontaneous acceptance of liver graft could be achieved in different mouse strains.^57^ Although other organs could induce tolerance at varying degrees,^58^ whole‐organ allografts of mouse heart and kidney could hardly achieve long‐term acceptance without immunosuppressive treatment. We conducted a signature gene analysis across three types of organ allograft models. Overlapping of signature DEGs indicates that 2872 genes are shared by three types of organ allografts (Figure 4A). Heart and kidney allografts had 250 overlapped signature genes, which covered the most of the unique genes in those two organs, respectively. In contrast, liver allografts have fewer overlapped genes with the other two organ allografts. We identified 357 uniquely expressed genes in liver allografts.

We calculated the ratio of integrated immune cells (Figure 4B). T cells_*Gzmb* ^+^ (‘***’*p* value < 0.001) and neutrophils_*Lcn2+* (‘***’*p* value < 0.001) demonstrated significant variation between liver and the other two organs. *Gzmb* expression is up‐regulated once after the activation of Tregs.^59^ *Lcn2* ^+^ neutrophils were contributed by liver allografts and ubiquitously expressed ribosomal genes. Ribosomal proteins are known to be associated with cellular metabolism and extra ribosomal activities such as inflammation in neutrophils.^60^ We observed enrichment of 40S ribosomal proteins such as *Rps2*, *Rps15*, *Rps12* and 60S ribosomal proteins including *Rpl8*, *Rpl10a*, *Rpl19* in this neutrophil subset, suggesting their activated state during reperfusion after liver allograft.^61^

Next, we compared DEGs in common immune cells between heart, kidney and liver allografts. We calculated the common DEGs (expressed in more than 5 cell types, *p* < 0.01) and most common DEGs (expressed in more than 20 cell types, *p* < 0.01). Common up‐regulated genes were associated with tolerance state, including previously described *Chil3*, *Saa3*, *Lcn2* and WAP domain proteins (Figure 4C). The most common DEGs were enriched in macrophages_*Chil3* ^+^, neutrophils_*Lcn2*+, T cells_*Gzmb* ^+^ and Kupffer cells (Figure S5G). *Chil3* and *Saa3* were also up‐regulated in stromal cells including hepatic stellate cells (Figure 4D).

Moreover, we performed KEGG pathway enrichment analysis of immune cells between heart, kidney and liver allografts (Figure 4E). Oxidative phosphorylation process was notably enriched in *H2‐Aa* ^+^ and *Clec4n* ^+^ M1 macrophages (*p* < 0.05) rather than *Chil3* ^+^ M2 macrophages. Oxidative phosphorylation plays an important role in macrophage activation and tolerance.^62^, ^63^ In liver allografts, we observed enrichment of complement and coagulation cascades in M1 macrophages, *Chil3* ^+^ M2 macrophages and *Lcn2* ^+^ neutrophils. Macrophages and neutrophils have been reported as a mediator of innate immunity to regulate inflammation and coagulation disorders in pig liver xenografts.^64^ Platelet activation was also enhanced in these cell types. Platelet activation and coagulation activation could positively regulate the long‐term graft survival.^65^ In mouse liver allografts, M1 macrophage_*Clec4n* ^+^ and M2 macrophage_*Chil3* ^+^ highly expressed coagulation factor X (*F10*), which is the key intermediate component in the conversion of thrombin during coagulation activation. *F10* also exhibited chemotactic capacity to promote M2 macrophage polarization.^66^ In this case, M1 and M2 macrophages have a more activated metabolic process in mouse liver allografts. They promoted coagulation activation to repair the tissue injury. The dynamic polarization of M1/M2 macrophages in mouse liver allografts may assist the long‐term tolerance induction.

### 3.6 Cross‐tissue interaction analysis of myeloid cells and lymphocytes in organ allografts

**FIGURE 5:**
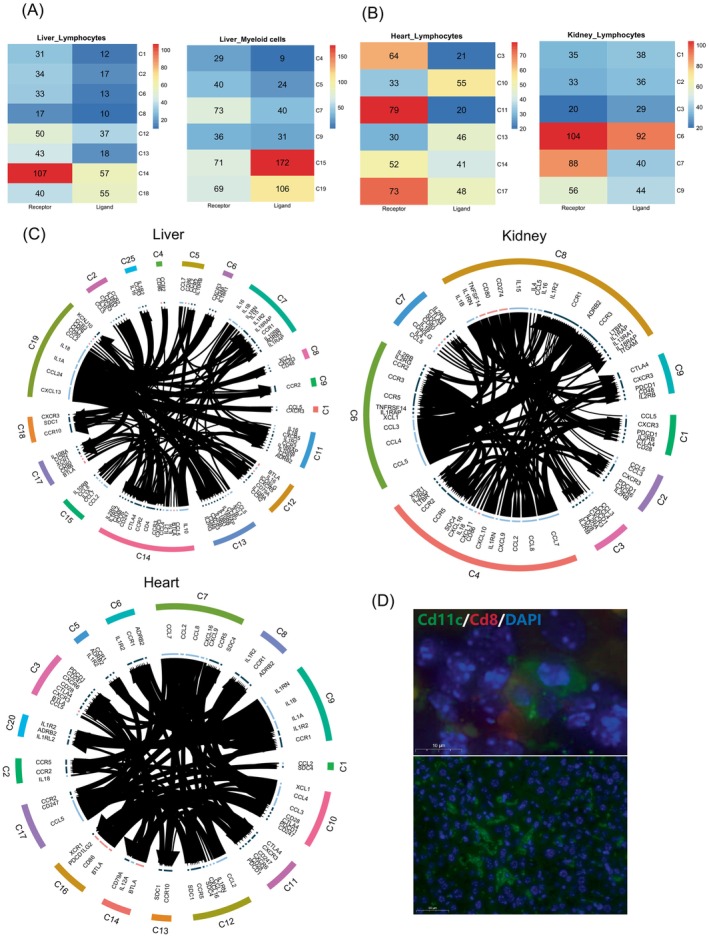
Ligand–receptor interactions in allografted organs. (A, B) The number of ligands and receptors involved in allografted liver (A) and the other two organs (B). (C) Ligand–receptor analysis of cytokines and immune checkpoint in the allografted liver, heart and kidney. Labels of clusters (C1: cluster1) represent the cell types in Figure 1C (D) Representative confocal immunofluorescence images of *Cd11c* and *Cd8* in liver allograft samples at day 7 post‐transplantation.

To characterize the regulatory networks across heart, kidney and liver allografts, we profiled ligand–receptors interactions in immune cells. In liver allografts, Tregs (Cluster 14, C14) contained the highest number of ligands and receptors (Figure 5A). In myeloid cells, Kupffer cells (C19) and monocytes_*Arg1* ^+^ (C15) harboured 106 and 172 ligands respectively. In heart lymphocytes, cytotoxic T‐cell_*Ms4a4b* ^+^ (C3) and proliferating T cells (C11) enriched a higher number of receptors than exhausted T cells (C10) (Figure 5B).

In liver allografts, Kupffer cells interacted with proliferating T cells, exhausted T cells, and Tregs via a series of chemokine and interleukin pathways (*IL1A‐IL1RAP*, *IL18‐IL18RAP*, *CCL24‐CCR2*, *CXCL13‐CXCR3*). Kupffer cells regulated multiple types of T cells including Tregs (Figure 5C). Tregs showed strong interactions with M1 macrophages, neutrophils, plasmacytoid dendritic cells and monocytes via the *IL10‐IL10RA/IL10RB* axis. Dendritic cells in liver allografts expressed high levels of *CD80/86* that bind to *CTLA‐4* in Tregs, suggesting that dendritic cells enhanced Tregs immunosuppressive activity via the *CTLA‐4* and *CD80/86* immune checkpoint pathways. Regulatory dendritic cells (*Cd11b+*) could secrete *IL10* and inhibit T cell proliferation as well as allograft rejection.^3^ We observed interactions between *Cd11b*+ dendritic cells and *Cd8*+ T cells in liver allografts (Figure 5D). The inhibition of T cell activation may reduce the interaction between T cells and hepatocytes and help to relieve the liver injury. In tumour microenvironment, *CD11b*+ dendritic cells with high expression levels of *IL23* and *TGF‐β* could induce *IL10+CD4* Tregs and promote tumour progression.^67^ *PD‐L1+* dendritic cells could suppress anti‐donor host T cell proliferation in mouse liver allograft.^2^ M1 macrophages and monocytes secrete several chemokines/interleukins (*CCL2*, *CCL7*, *CCL8*, *IL1A*, *IL1B*, *ILRN*) that bind to cytotoxic T cells, M2 macrophages and neutrophils. Myeloid cells such as M1/M2 macrophages and neutrophils suppressed T cell activity in liver allografts through the interaction with Tregs. Compared with the rejection state in heart and kidney allograft, regulatory dendritic cells, Kupffer cells, macrophages and Tregs in liver allografts reshaped an immunosuppressive microenvironment.

## 4 DISCUSSION

Recent advance in pig‐to‐human kidney xenotransplantation has achieved normal graft function for at least 1 week.^68^ The immune response after pig‐to‐human kidney xenotransplantation has also been studied using multimodal immunophenotyping including gene expression profiling and gene expression profiling.^69^ Different from clinical organ transplantation in which the immunosuppressant is involved, the mouse organ transplantation models could provide a platform to simply concentrate in the immune response after transplantation. These models have become a powerful tool to elucidate the mechanisms by which the liver induces donor‐specific tolerance whereas heart and kidney transplantation leads to allograft rejection. The organ transplantation models could help to facilitate treatment to induce acceptance of solid organ allografts in the clinic.^70^, ^71^, ^72^, ^73^

In this work, we constructed the mouse models of liver, heart and kidney allografts. We characterized the cell composition and immune microenvironment in the acute rejection state (heart, kidney) and tolerance state (liver) at day 7 post‐transplantation. The cellular and molecular mechanisms underlying allograft rejection/tolerance were initially deciphered. The death by neglect of intrahepatic activated T cells and coinhibitory pathways in *CD8*+ T cells were reported to be associated with the early phase of liver allograft tolerance.^74^ High proportions of proliferating T cells and exhausted T cells were identified in liver allografts. Early induction of T cell exhaustion reshapes the T cell composition in liver allografts. A high proportion of ribosomal gene expressing T cells (T cells_Rp high) in liver allografts may affect the Tregs activation and trigger the T cells exhaustion process.^75^

The metabolic homeostasis in allografted liver was perturbed after transplantation. The Nr1h3‐Cebpb‐Bhlhe40 complex that regulates the insulin receptor signalling pathway is selectively expressed in liver allografts. This complex could affect T‐cell proliferation as well as IFNγ production through insulin secretion regulation.^55^, ^56^ Decreased insulin signalling was identified in another scRNA‐seq study of mouse liver transplantation from day 7 to day 15 post‐transplantation.^9^ However, bulk RNA‐seq of allografted mouse liver showed increased insulin secretion and fatty acid process at day 60 post‐transplantation, suggesting a restorative metabolic homeostasis in long‐term immune tolerance state.

Some studies have indicated that Tregs could predominantly contribute to the long‐term tolerance in liver allografts.^3^, ^76^ Consistently, we found that Tregs and signature genes such as *Ctsc*, *Cebpe* and *Ctla4* were enriched at day 7 post‐transplantation in both scRNA‐seq and bulk RNA‐seq data. KEGG pathway enrichment suggested antigen processing and presentation program, cytokine‐receptor interaction between myeloid cells and T cells through the response to interferon‐gamma and the Jak–STAT signalling pathway. In bulk RNA‐seq datasets, gene function enrichment at day 14 post‐transplantation involved cell killing, tolerance induction and regulation of interferon‐gamma production. KEGG pathway enrichment of T helper cell differentiation indicated a strong tolerance induction stage at day 14 post‐transplantation. Enrichment of the interferon‐gamma‐mediated signalling pathway and myeloid leukocyte activation at day 28 post‐transplantation promote immunoregulatory interactions between lymphoid and nonlymphoid cells. Positive regulation of insulin secretion is associated with cellular response to glucose stimulus. Insulin secretion, metabolic process regulation and myeloid cell interactions demonstrated a high correlation between scRNA‐seq and bulk RNA‐seq at day 7 post‐transplantation.

The dynamic picture of spontaneous tolerance induction process in mouse liver allografts illustrated distinctive regulatory patterns between myeloid cells and T cells. Macrophages have been reported to be associated with innate immune responses in organ allografts.^77^ Kupffer cells could directly interact with Tregs to stimulate their proliferation. Secretion of IL‐10 in Kupffer cells could also inhibit the activation of cytotoxic T cells.^78^, ^79^ Studies have shown that Kupffer cells with high expression of PD‐L1 can inhibit the proliferation and functions of T cells.^80^ Moreover, two critical pathways (platelet activation and coagulation activation of oxidative phosphorylation) participated in the polarization of M1/M2 macrophages and Kupffer cell function. Kupffer cells with M2‐like protective phenotypes have also been observed in the other scRNA‐seq study of mouse liver transplantation.^9^ Enhancement of these pathways contributed to the tissue protection and long‐term tolerance. It has been reported that the microenvironment in liver could help the differentiation of regulatory DCs, which can secrete IL‐10 to inhibit T cell response and promote regulatory T cell generation.^3^, ^67^ We identified *Cd11b*+ regulatory dendritic cells in liver allografts and observed their interaction with *Cd8*+ T cells. In heart and kidney allografts, enrichment of the HIF‐1 signalling pathway revealed the potential hypoxic tissue injury which could accelerate acute rejection. Enrichment of the prolactin signalling pathway and PI3K–Akt signalling pathway were observed in heart and kidney allografts. Prolactin may cooperate with proinflammatory stimuli and promote tissue fibrosis, which could accelerate acute rejection in kidney allografts.^81^, ^82^, ^83^ Clinically, there are no reliable immunological indicators that enable patients to be weaned from immunosuppressants without the risk of rejection. The correlation between mouse liver allografts and PBMCs in T cells and macrophages provides clues for further biomarkers screening to evaluate the allograft tolerance state.

In conclusion, we generated single‐cell transcriptome datasets of mouse solid organ allografts. We resolved the immune microenvironment in organ allografts and analysed the molecular interaction between myeloid cells and lymphocytes. One limitation of our study is the cell type bias. Incomplete parenchymal cell types hindered interaction analysis between parenchymal cells and immune cell to evaluate the tissue injury state and perturbation of organ functions. Several studies have reported the scRNA‐seq profiling of human rat, and mouse liver transplantation to explore the tissue injury and potential therapeutic targets.^9^, ^84^, ^85^ We observed similar perturbation of metabolic homeostasis and protective phenotypes of Kupffer cells. The other limitation is the lack of B cells in kidney allografts day 7 post‐transplantation. It has been reported that kidney transplant operational tolerance patients (stable graft function without any immunosuppressive drugs for more than 1 year) demonstrated increasement of immature B cells.^86^ A single cell survey of mouse kidney transplantation using mismatched donor‐recipient strain combinations compared the transcriptome between accepted and rejected renal graft.^87^ This study revealed a shifting from a T cell‐dominant to a B cell‐rich population by 6 months. B cells were also the early infiltrating cells in accepted model rather than rejected model. Contradictory role of B cell subsets was identified between protective function and humoral immunity mediated rejection. However, a similar scRNA‐seq study of mouse kidney transplantation (BALB/c donors and C57Bl6/J recipients) demonstrated a very low fraction of B cells.^10^ A sharp reduction of B cells was observed from day 7 to day 15 post‐transplantation (7.85%–1.38%). Thus, we reasoned that in the early stage of kidney transplantation, infiltration of B cell is hard to profile using different scRNA‐seq platforms. Infiltrated T cells account for the vast majority proportion of lymphocytes. Richest ligand and receptor pairs (chemokines) were also observed in macrophages. Common signatures of proinflammatory macrophage contributed early kidney allograft inflammation in acute rejection.^88^ Nevertheless, resources in this study could be used to gain further insights into biological questions in organ allografts.

## AUTHOR CONTRIBUTIONS

All authors contributed to the study conception and design. Yingying Wang, Jun Pan, Fang Ye, Xiaoming Meng and Guoji Guo conceived and designed the experiments; Yingying Wang, Hui Li, Haide Chen and Junqing Wu performed the experiments; Fang Ye, Jun Pan, Chengxuan Yu, Yanyu Xiao, Lijiang Fei and Jiaqi Li analysed the data; Fang Ye, Yingying Wang and Jun Pan wrote the paper; Jun Pan and Yingying Wang provided funding and supervision. Yijun Wu and Jiajia Mao revised the paper. All authors read and approved the final manuscript.

## FUNDING INFORMATION

This study was supported by grants from the National Natural Science Foundation of China (81800658), Zhejiang Medical Science and Technology Projects (2019330597 and 2019330585). The establishment of mouse models of solid organ transplantation was supported by Heymouse Biological Technology Co., Ltd.

## CONFLICT OF INTEREST STATEMENT

The authors declare no competing interests.

## Supporting information


**Figure S1.** Data quality of single cell RNA‐seq data. (A) Pictures of liver, heart and kidney allografts and representative HE staining images of allografted tissues. (B) Number of genes and transcripts (unique molecular identifiers, UMIs) in different samples after quality control (PBMCs: peripheral blood mononuclear cells, TILs: Tissue resident lymphocytes). (C) Distribution of different samples in merged datasets. (D) Violin plot showing the expression of cell type specific marker in Figure 1C. The number represents different clusters.

**Figure S2.** Gene function analysis of bulk RNA‐seq data. Gene ontology enrichment (A) and KEGG pathway enrichment analysis (B) of differentially expressed genes in bulk RNA‐seq data at different time point after liver transplantation.

**Figure S3.** Re‐clustering of lymphocytes in allografted liver. (A) Dot plots showing the cell type specific marker genes of major immune cells in allografted liver. (B) UMAP plot showing the re‐clustering of T cell subsets in allografted liver. (C) Heatmap showing differentially expressed genes in T cell subsets in grafted liver. (D) Volcano plot showing the differentially expressed genes between Tregs and other T cell subsets. (Gene coloured in red: up‐regulated in Treg; Gene coloured in blue: down regulated in Treg). avg_logFC in *x*‐axis represent the average log fold changes. −log10 (*p*_val_adj) in *y*‐axis represent the −log_10_ adjusted *p* values. (E) Heatmap showing differentially expressed cell cycle genes in T cell subsets in grafted liver. Each row represents a cell cycle gene. S.score represents the cell cycle gene expression level in S phase. G2M.score represents the cell cycle gene expression level in G2/M phase. (F) t‐Distributed Stochastic Neighbour Embedding (t‐SNE) plot showing the re‐clustering results (up) and tissue distribution (below) of T cell clusters. (G) The fraction of T cell subsets in allografted liver and the other two organs (nonLiver). (H) The fraction of T cell subsets in grafted liver compare with grafted heart and kidney (nonLiver). The rows represent the cluster of T cell subsets in (F). (I) Volcano plot showing the differentially expressed genes in T cells between allografted liver and the other two organs (heart and kidney).

**Figure S4.** Re‐clustering of myeloid cells in allografted liver. (A) Top 50 cytokines of Kupffer cells in allografted liver. Cluster labels (C1: cluster1) represent the liver cell types in Figure 1C. (B) Ligand–receptor analysis of *IL10* in Kupffer cells. (C) Re‐clustering of macrophages subsets, monocytes and Kupffer cells in allografted liver. (D) Heatmap showing differentially expressed genes in macrophages, monocytes and Kupffer cells. (E) Volcano plot showing the differentially expressed genes between *Chil3+* macrophages and other macrophage subsets. avg_logFC in *x*‐axis represent the average log fold changes. −log10(*p*_val_adj) in *y*‐axis represent the −log_10_ adjusted *p* values (not significant: NoSinifi). (F) Ligand–receptor analysis of *CSF1R* in Kupffer cells.

**Figure S5.** Gene modules and function enrichment of immune cells in normal and allografted moues liver. (A) Dendrogram of gene modules in grafted liver. Gene module is colour‐coded. (B) Gene adjacency heatmap showing the correlation of gene modules in allografted liver. The colour scale is based on a correlation score from 0 (blue) to 1(red). (C–F) Gene ontology enrichments of top 20 up‐regulated transcription factors in B cells (C), dendritic cells (D), macrophages (E) and T cells (F) between allografted and normal liver (TP: modules in allografted liver data, MCA: modules in mouse cell atlas normal liver data). (G) Volcano plot showing the most common differentially expressed genes in major immune cells between allografted liver (tolerance state) and the other two organs (rejection state). Different immune cells are colour‐coded.

**Table S1.** Different gene modules in WGCNA analysis.

**Table S2.** Modules and transcription factors in normal mouse liver.

**Table S3.** Modules and transcription factors in allografted mouse liver.

## Data Availability

The raw sequence data and processed data of single cell RNA‐seq have been deposited in the Gene Expression Omnibus database (GSE189069). Bulk RNA‐seq data have been deposited in figshare (https://figshare.com/s/1e76ace9c48ab42907d1). Example scripts to process and analyse data can be found on the following website (https://github.com/ggjlab/mca_data_analysis). Detailed information is available from the corresponding author upon reasonable request.
