# Supplementary figures and images for: Dissecting the immune discrepancies in mouse liver allograft tolerance and heart/kidney allograft rejection
Source: Cell Prolif. 2023 Sep 25;57(3):e13555. doi: 10.1111/cpr.13555 (PMC10905343; doi:10.1111/cpr.13555)

(A)

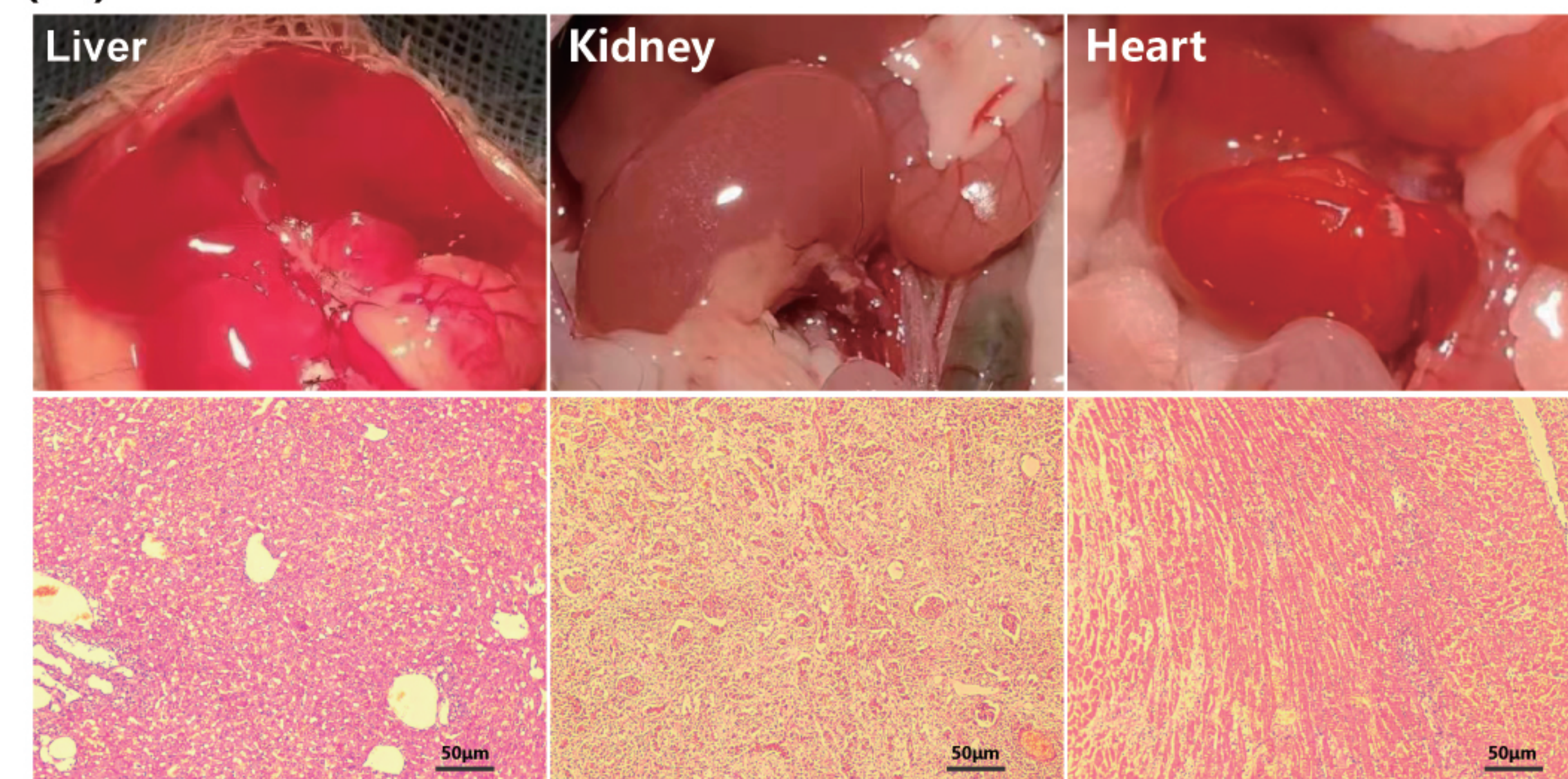

(B)

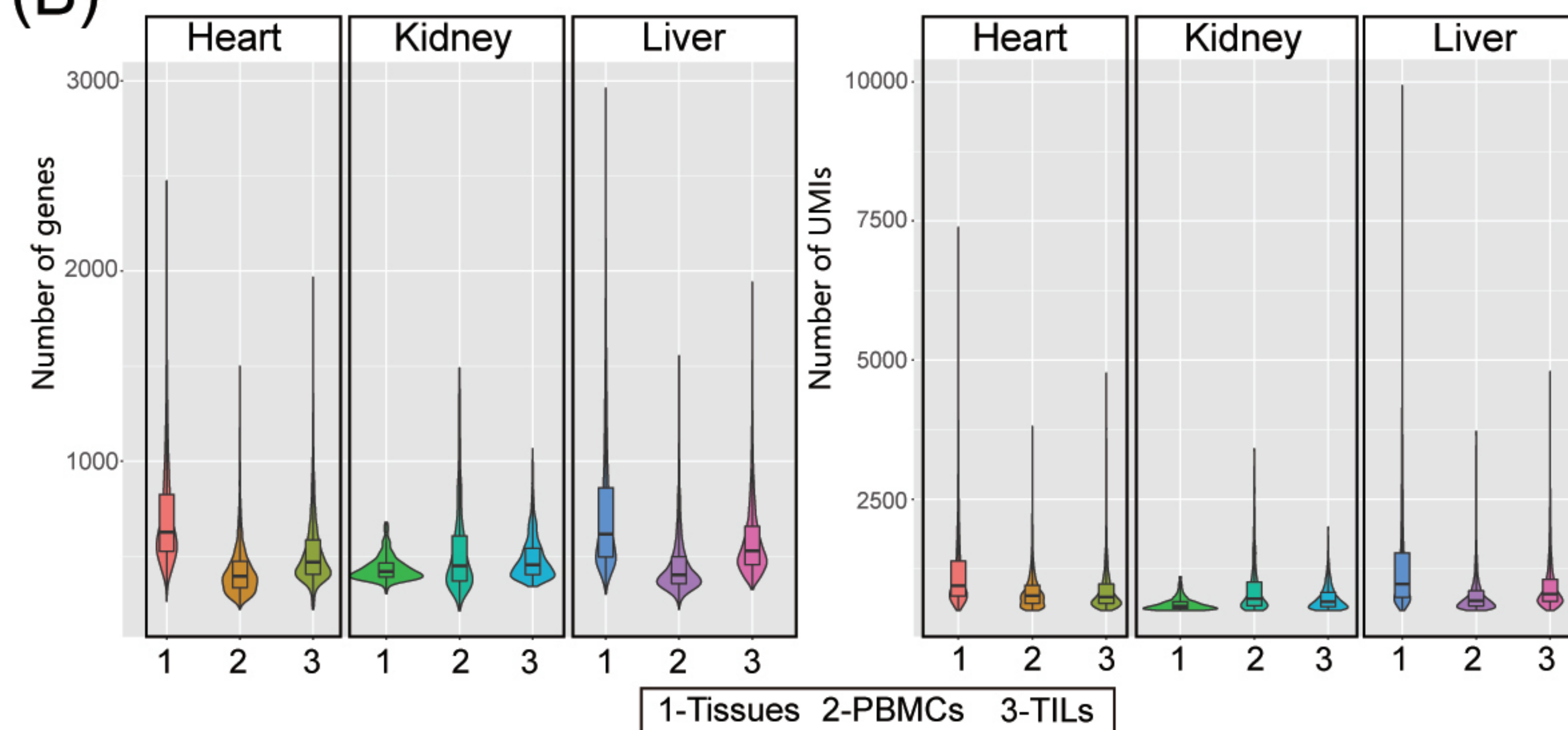

(C)

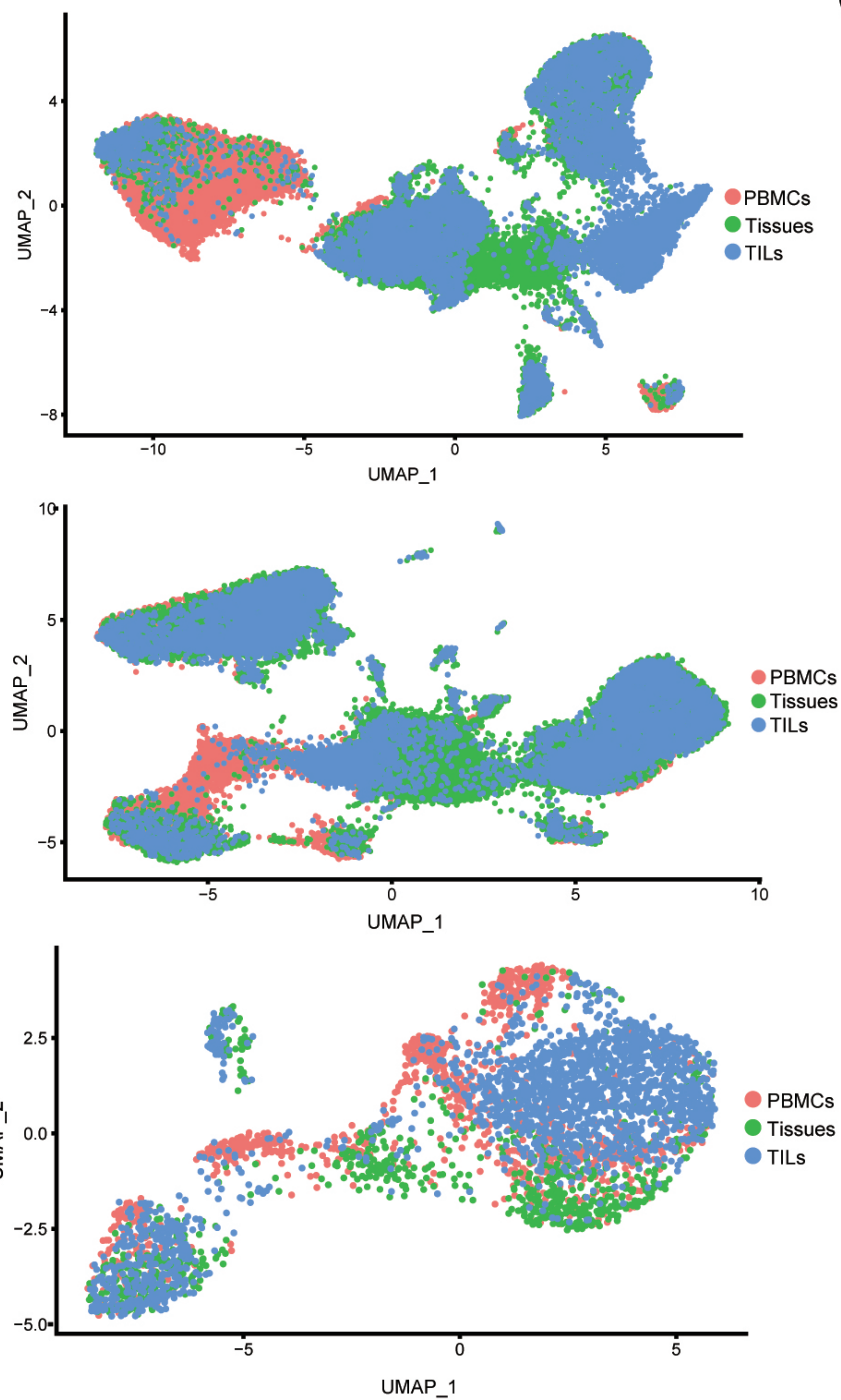

(D)

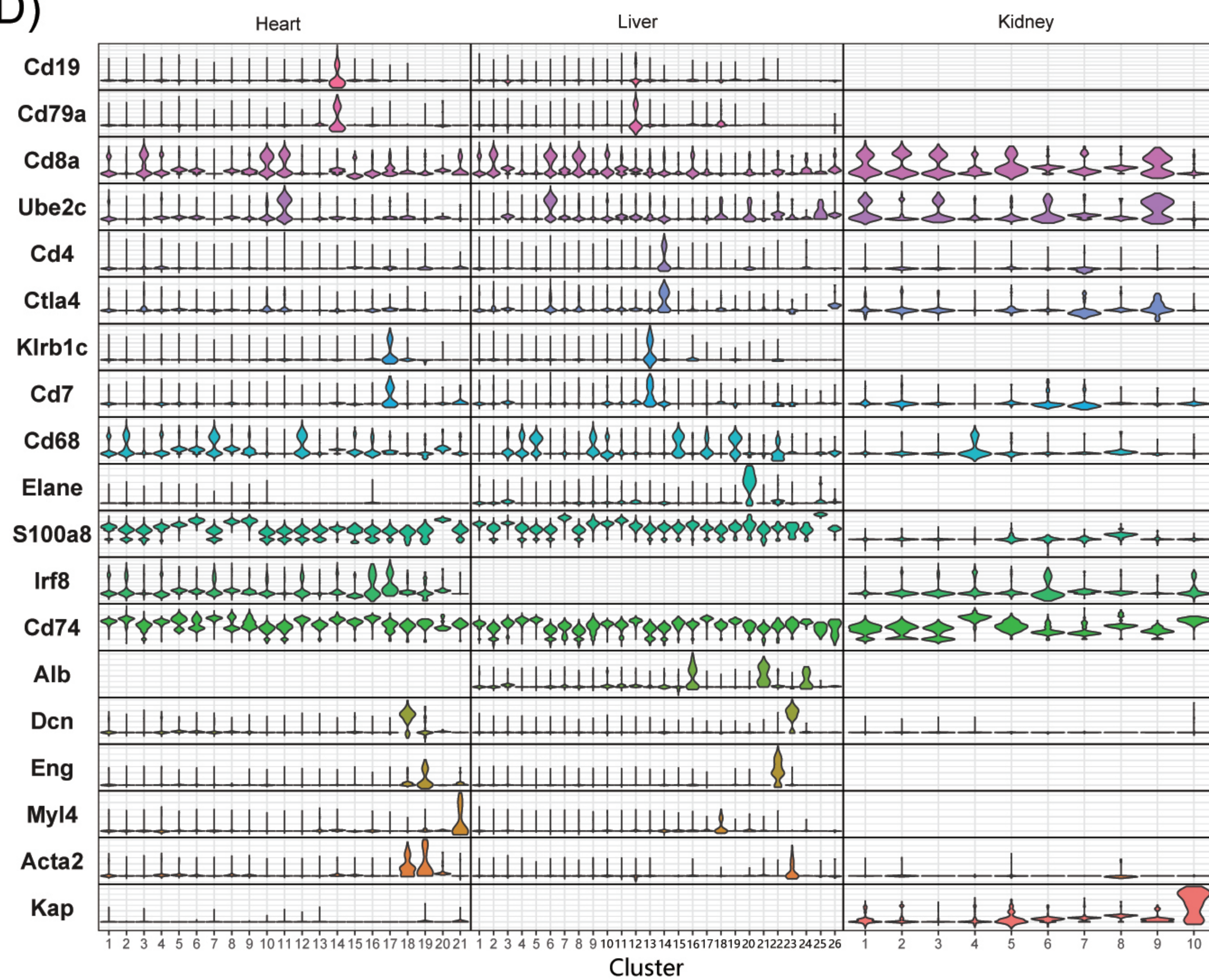

Supplement: Supplementary file 1 — Figure S1. Data quality of single cell RNA‐seq data. (A) Pictures of liver, heart and kidney allografts and representative HE staining images of allografted tissues. (B) Number of genes and transcripts (unique molecular identifiers, UMIs) in different samples after quality control (PBMCs: peripheral blood mononuclear cells, TILs: Tissue resident lymphocytes). (C) Distribution of different samples in merged datasets. (D) Violin plot showing the expression of cell type specific marker in Figure 1C. The number represents different clusters. [file CPR-57-e13555-s008.pdf]

(A)

Day7

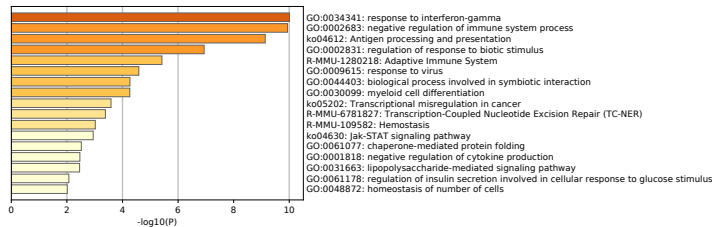

Day14

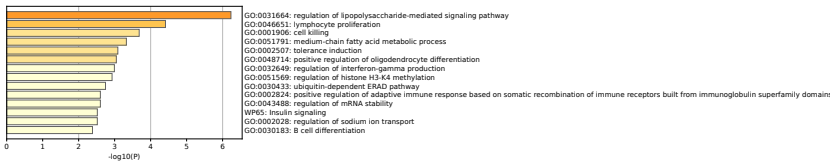

Day28

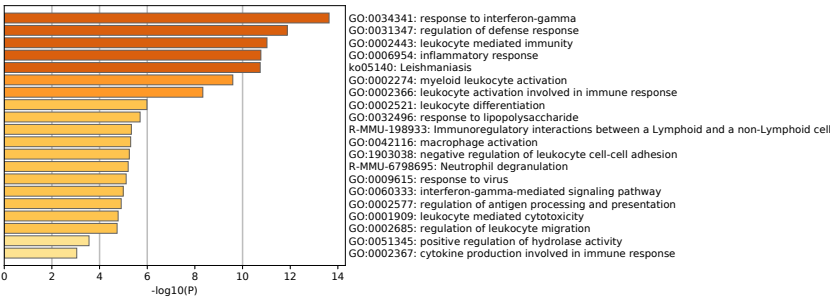

Day60

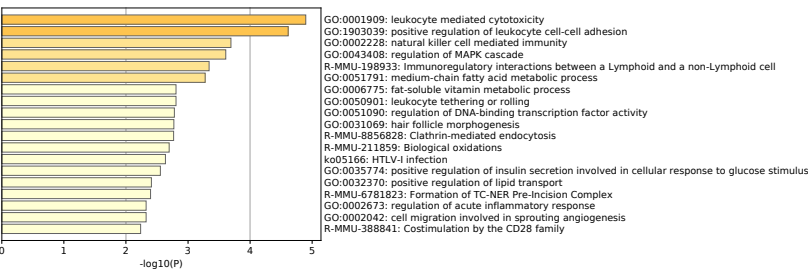

(B)

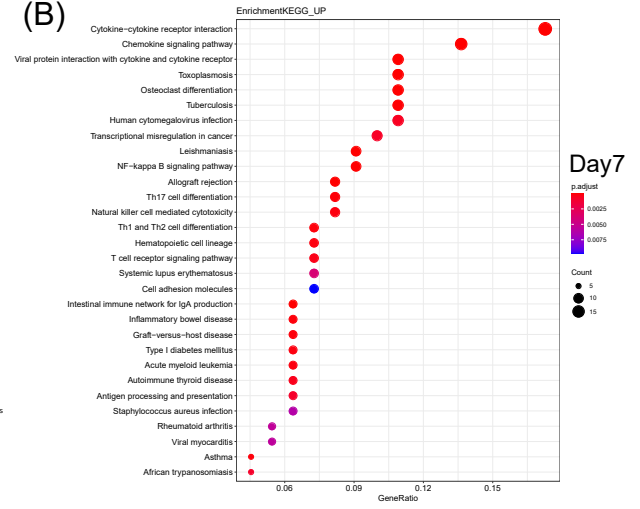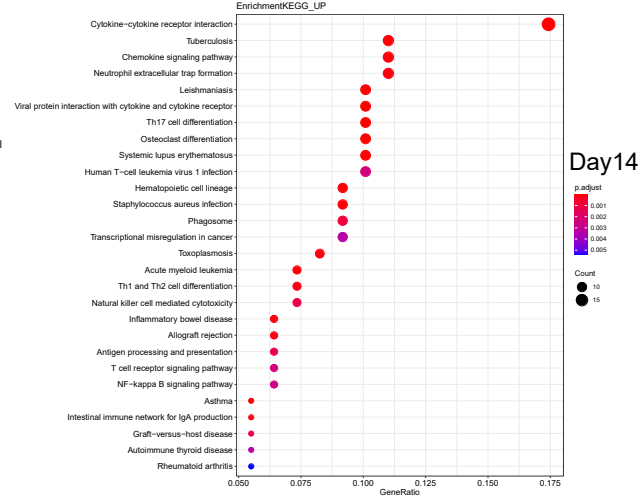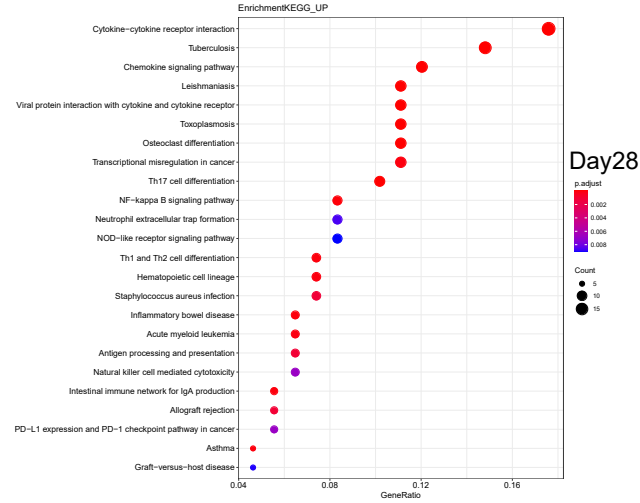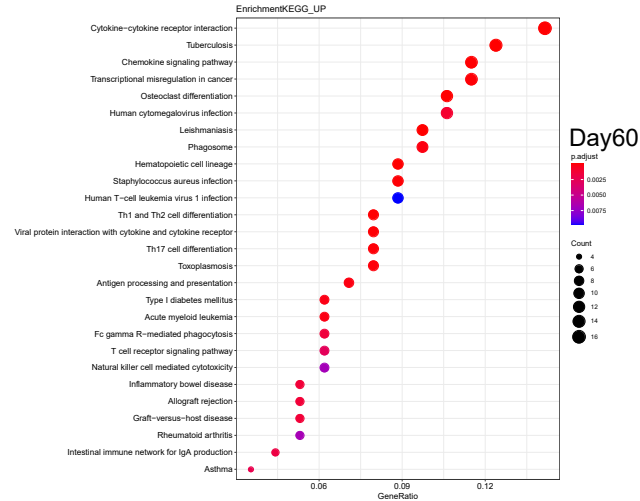

Supplement: Supplementary file 2 — Figure S2. Gene function analysis of bulk RNA‐seq data. Gene ontology enrichment (A) and KEGG pathway enrichment analysis (B) of differentially expressed genes in bulk RNA‐seq data at different time point after liver transplantation. [file CPR-57-e13555-s001.pdf]

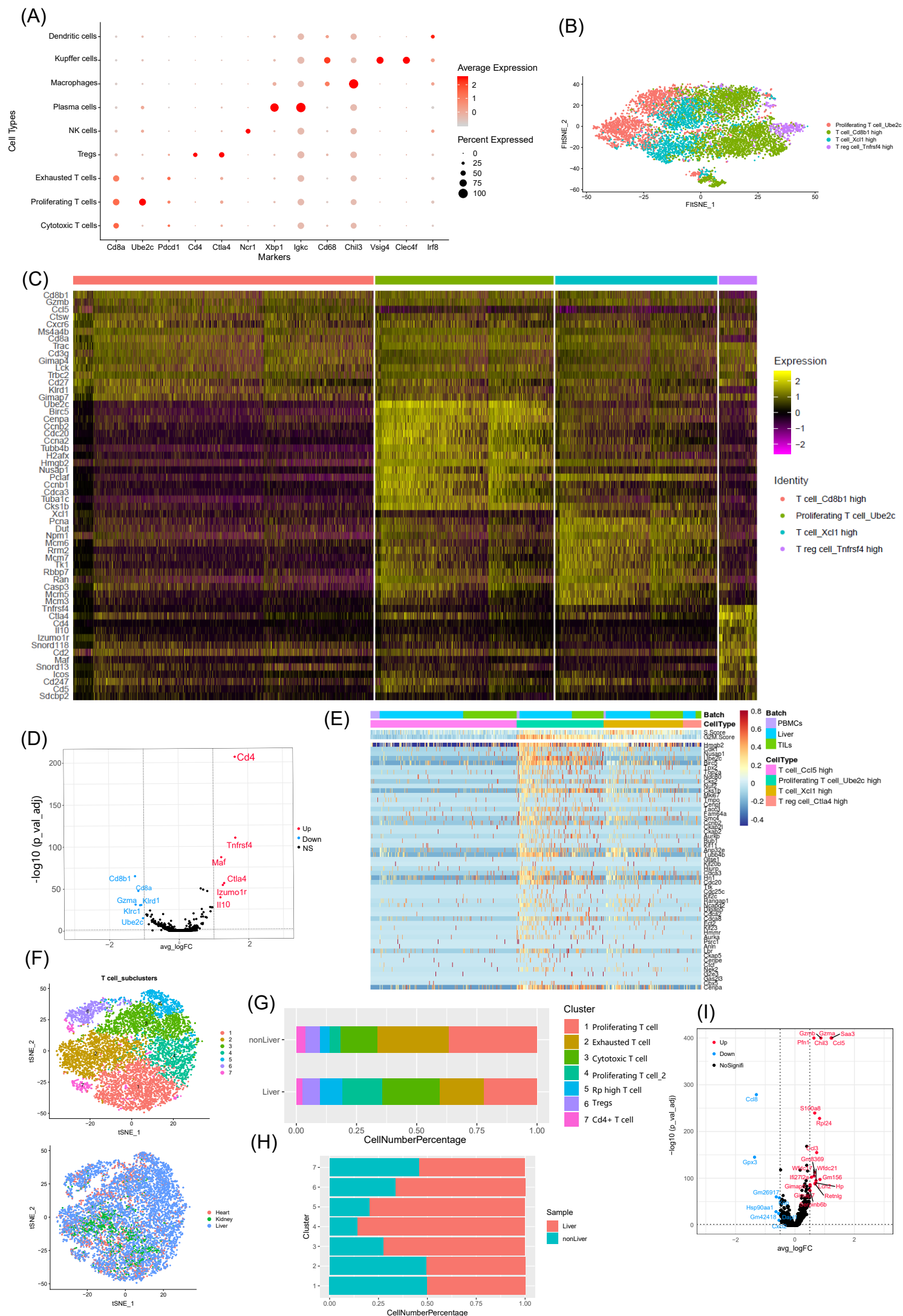

Supplement: Supplementary file 3 — Figure S3. Re‐clustering of lymphocytes in allografted liver. (A) Dot plots showing the cell type specific marker genes of major immune cells in allografted liver. (B) UMAP plot showing the re‐clustering of T cell subsets in allografted liver. (C) Heatmap showing differentially expressed genes in T cell subsets in grafted liver. (D) Volcano plot showing the differentially expressed genes between Tregs and other T cell subsets. (Gene coloured in red: up‐regulated in Treg; Gene coloured in blue: down regulated in Treg). avg_logFC in x‐axis represent the average log fold changes. −log10 (p_val_adj) in y‐axis represent the −log10 adjusted p values. (E) Heatmap showing differentially expressed cell cycle genes in T cell subsets in grafted liver. Each row represents a cell cycle gene. S.score represents the cell cycle gene expression level in S phase. G2M.score represents the cell cycle gene expression level in G2/M phase. (F) t‐Distributed Stochastic Neighbour Embedding (t‐SNE) plot showing the re‐clustering results (up) and tissue distribution (below) of T cell clusters. (G) The fraction of T cell subsets in allografted liver and the other two organs (nonLiver). (H) The fraction of T cell subsets in grafted liver compare with grafted heart and kidney (nonLiver). The rows represent the cluster of T cell subsets in (F). (I) Volcano plot showing the differentially expressed genes in T cells between allografted liver and the other two organs (heart and kidney). [file CPR-57-e13555-s004.pdf]

(A)

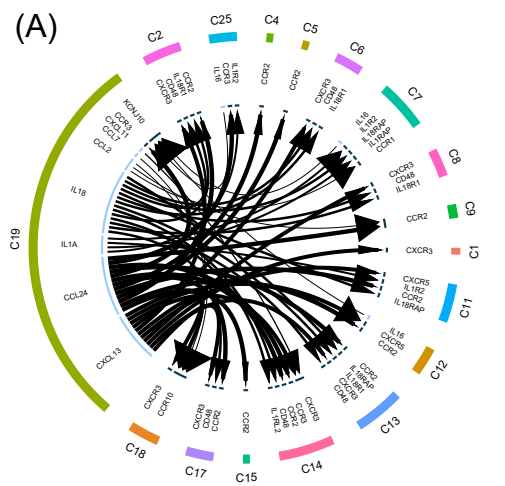

(B)

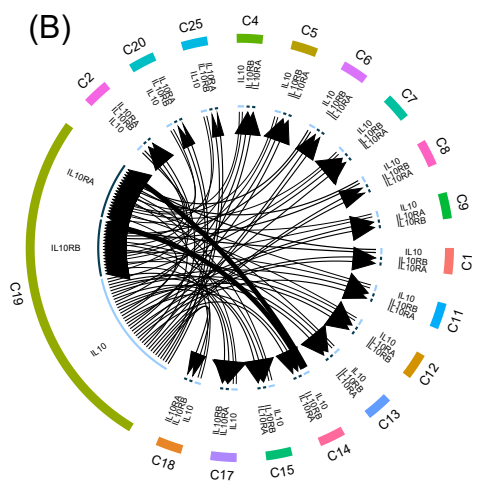

(C)

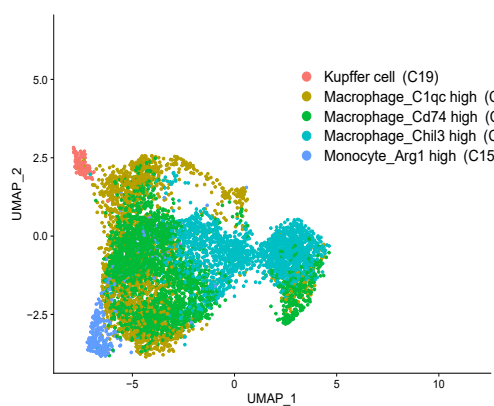

(D)

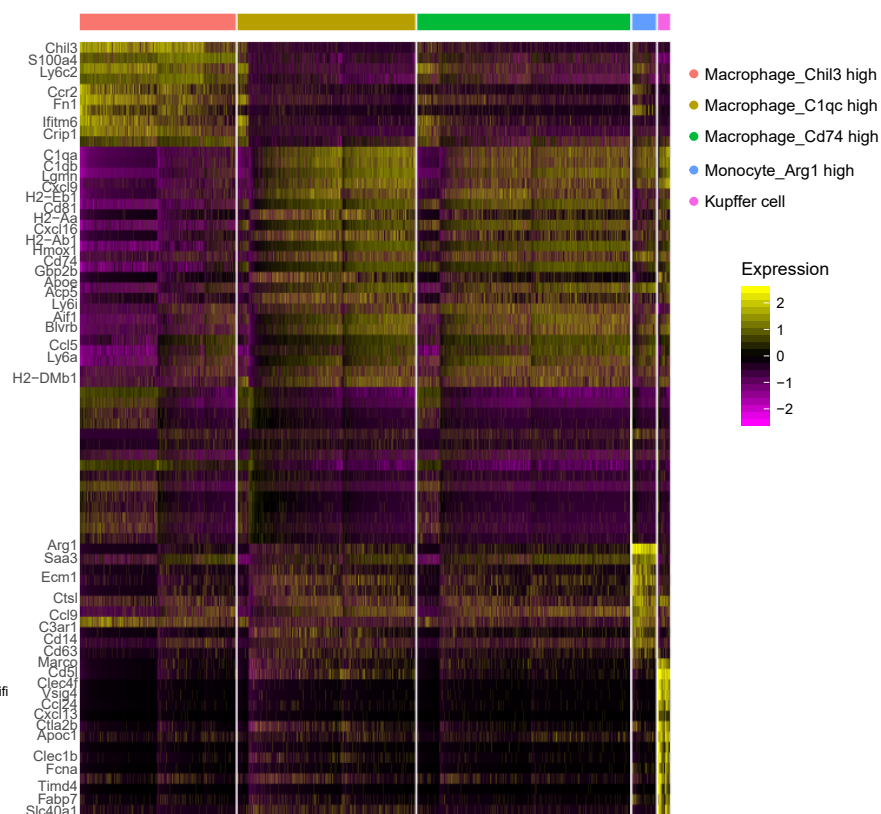

(E)

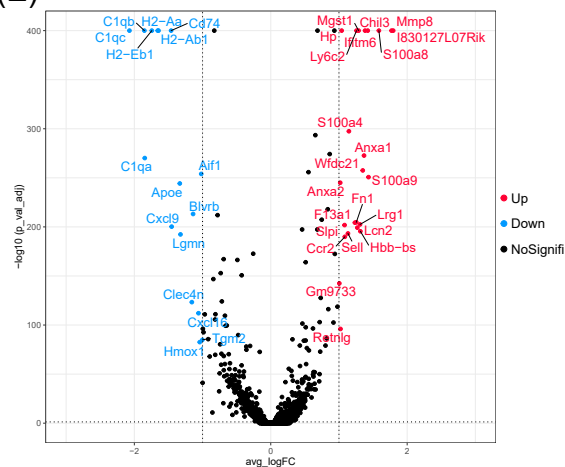

(F)

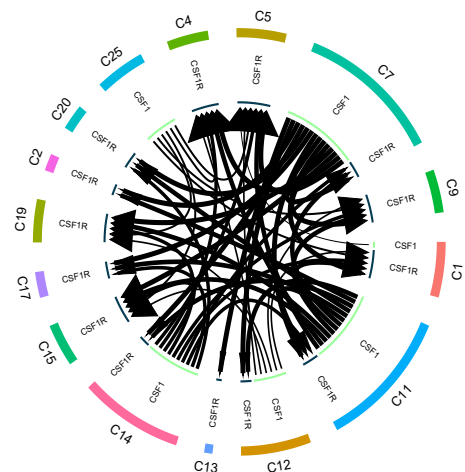

Supplement: Supplementary file 4 — Figure S4. Re‐clustering of myeloid cells in allografted liver. (A) Top 50 cytokines of Kupffer cells in allografted liver. Cluster labels (C1: cluster1) represent the liver cell types in Figure 1C. (B) Ligand–receptor analysis of IL10 in Kupffer cells. (C) Re‐clustering of macrophages subsets, monocytes and Kupffer cells in allografted liver. (D) Heatmap showing differentially expressed genes in macrophages, monocytes and Kupffer cells. (E) Volcano plot showing the differentially expressed genes between Chil3+ macrophages and other macrophage subsets. avg_logFC in x‐axis represent the average log fold changes. −log10(p_val_adj) in y‐axis represent the −log10 adjusted p values (not significant: NoSinifi). (F) Ligand–receptor analysis of CSF1R in Kupffer cells. [file CPR-57-e13555-s002.pdf]

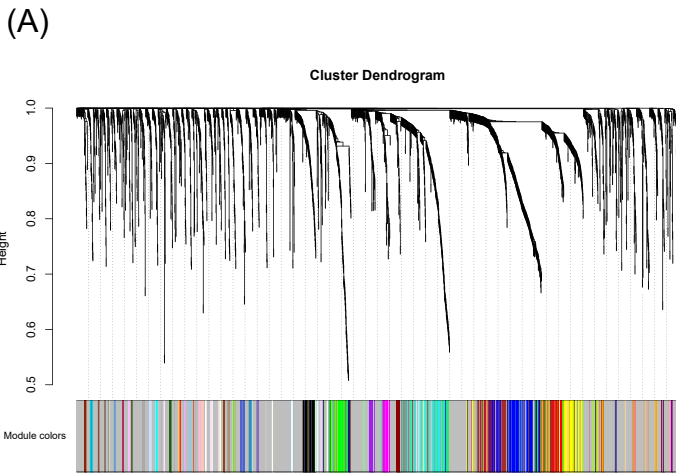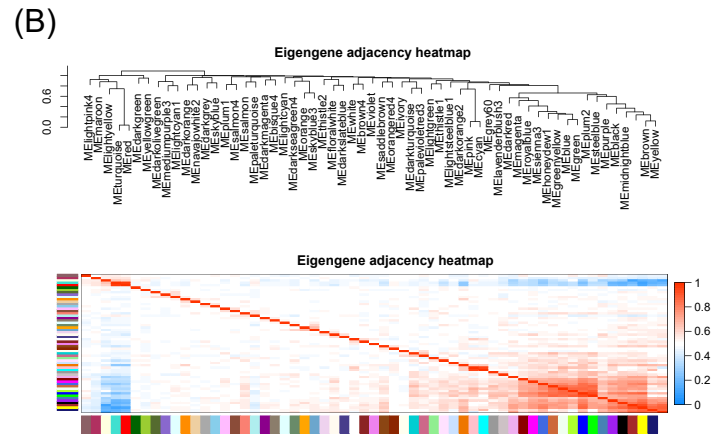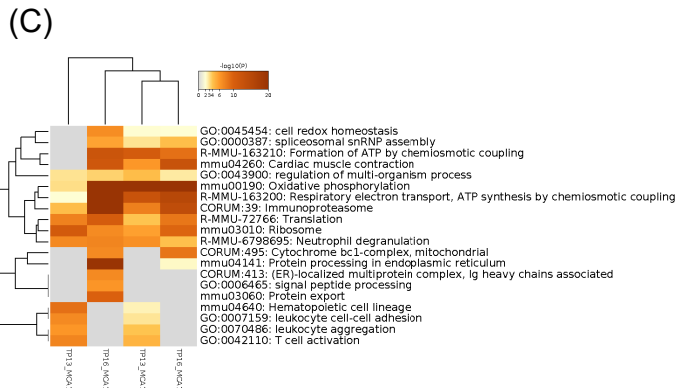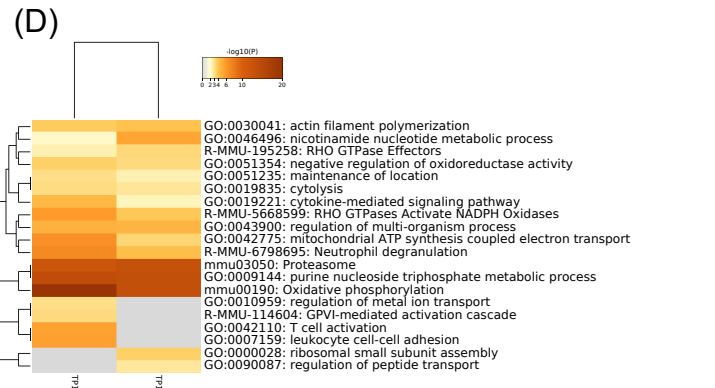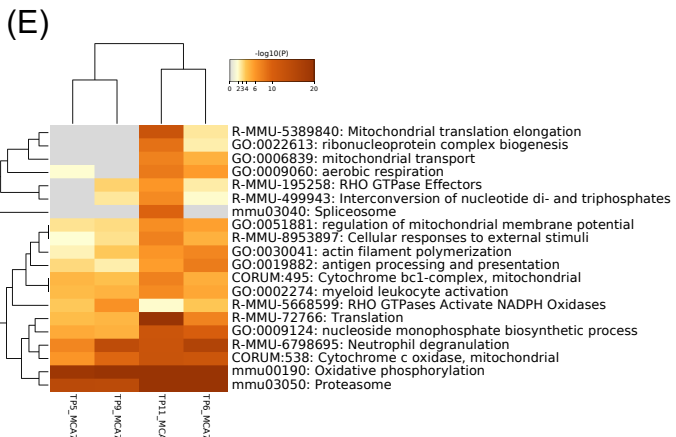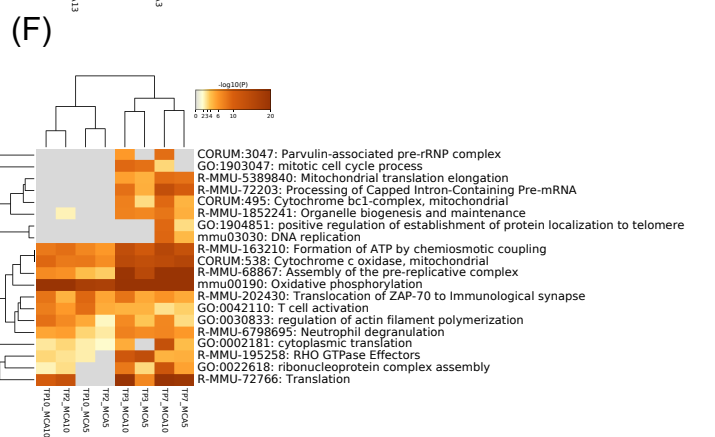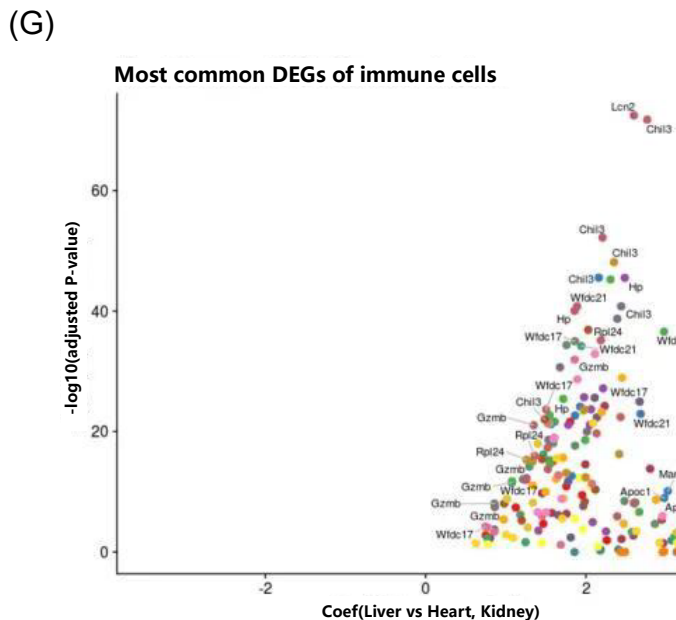

Supplement: Supplementary file 5 — Figure S5. Gene modules and function enrichment of immune cells in normal and allografted moues liver. (A) Dendrogram of gene modules in grafted liver. Gene module is colour‐coded. (B) Gene adjacency heatmap showing the correlation of gene modules in allografted liver. The colour scale is based on a correlation score from 0 (blue) to 1(red). (C–F) Gene ontology enrichments of top 20 up‐regulated transcription factors in B cells (C), dendritic cells (D), macrophages (E) and T cells (F) between allografted and normal liver (TP: modules in allografted liver data, MCA: modules in mouse cell atlas normal liver data). (G) Volcano plot showing the most common differentially expressed genes in major immune cells between allografted liver (tolerance state) and the other two organs (rejection state). Different immune cells are colour‐coded. [file CPR-57-e13555-s007.pdf]
